# Recent developments in gold-catalyzed cycloaddition reactions

**DOI:** 10.3762/bjoc.7.124

**Published:** 2011-08-09

**Authors:** Fernando López, José L Mascareñas

**Affiliations:** 1 Instituto de Química Orgánica General, CSIC, C/ Juan de la Cierva 3, 28006, Madrid, Spain, (+) 34 915622900, Fax: (+) 34 915644853; 2 Departamento de Química Orgánica, Centro Singular de Investigación en Química Biológica y Materiales Moleculares, y Unidad Asociada al CSIC. Universidad de Santiago de Compostela, Avda. de las Ciencias, s/n, 15782, Santiago de Compostela, Spain, (+) 34 981563100 Fax: (+) 34 981595012

**Keywords:** alkene, alkyne, allene, catalysis, cycloaddition, gold

## Abstract

In the last years there have been extraordinary advances in the development of gold-catalyzed cycloaddition processes. In this review we will summarize some of the most remarkable examples, and present the mechanistic rational underlying the transformations.

## Introduction

In modern organic synthesis, the criteria of efficiency, versatility, economy and ecology are of paramount importance [1]. Consequently, nowadays there is an increasing demand for the development of methods and strategies that allow the transformation of readily available precursors into target-relevant products in a rapid, economical and efficient manner. Cycloaddition reactions are among the synthetic tools that best fit these criteria, because by allowing the generation of at least two bonds and one cycle in a single operation, they produce a rapid increase in skeletal and also stereochemical complexity, and this is usually beneficial in terms of shortening the access to polycyclic products. Importantly, they generally involve the simple addition of two or more molecules, thereby being atom economical, and take place with high regio- and stereocontrol [2–3]. Unfortunately, the realm of classical cycloaddition reactions is relatively small and restricted to precursors presenting suitable electronic properties. In this regard, transition metal complexes, owing to their multiple coordination and activation properties, offer excellent opportunities for the discovery of new cycloaddition alternatives, and in many cases they can be used in a catalytic manner.

Although transition metal-catalyzed cycloadditions have been known since the mid-20th century, it was not until the 80s and 90s that they were recognized as versatile and powerful synthetic tools. So far, most examples of transition metal-catalyzed cycloadditions have involved the use of rhodium, ruthenium, cobalt, nickel or palladium catalysts [4]. In recent years, however, platinum and particularly gold complexes have also emerged as excellent catalysts for the promotion of novel types of cycloaddition reactions, usually involving non-activated unsaturated systems (e.g., alkynes, allenes, alkenes or 1,3-dienes) [5–12].

Today it is well known that the excellent reactivity of these group-11 catalysts can in part be explained in terms of relativistic effects, particularly marked in the case of gold [13–14], that induce the contraction of 6s and the expansion of 5d orbitals. As a consequence this metal exhibits singular characteristics, such as a high carbophilicity, affinity for π-unsaturated systems (e.g., alkynes, alkenes or allenes), and a low propensity to participate in typical redox processes characteristic of other transition metal catalytic cycles (e.g., oxidative additions and reductive eliminations). Therefore, platinum(II) and particularly gold(I) or (III) complexes tend to activate alkynes, alkenes or allenes in a highly chemoselective manner; activation that opens interesting reaction pathways that usually involve carbocationic intermediates. Also very important is the possibility of modulating the properties of the metal through modification of its ancillary ligands (e.g., phosphines, *N*-heterocyclic carbenes, etc.), which considerably widens the potential and versatility of these catalysts, and in particular of those consisting of cationic gold(I) complexes (e.g., ligand–Au^+^).

In this context, a number of research groups have in recent years embarked on the design and development of new cycloaddition reactions promoted by gold(I) and (III) catalysts, and hence the field has experienced a remarkable expansion. In the following section, we summarize some of the most recent contributions, organized according to the type of unsaturated system that is initially activated by the electrophilic gold complex [12].

Related Pt-catalyzed cycloadditions will only be mentioned when required in the context of a particular gold-catalyzed process, or to highlight the differences between these carbophilic catalysts. On the other hand, dipolar cycloaddition reactions in which the gold complex does not activate π-bonds, but rather behaves as a more conventional Lewis acid, are not discussed [15–17].

## Review

### Cycloadditions initiated by gold-activation of alkynes

Many examples of homogeneous catalysis employing gold complexes exploit the ability of these metal catalysts to bind chemoselectively to C–C triple bonds, promoting a subsequent attack of a nucleophile on these activated unsaturated systems. Although these metal carbophilic catalysts also coordinate to alkenes, dienes and allenes in a similar way [18–22], nucleophiles seem to have a kinetic preference for attacking activated alkynyl systems, which warrants high chemoselectivities.

Based on this reactivity pattern, several groups have demonstrated that certain substrates containing an alkyne and a carbonyl unit can be transformed, under Au(I/III) catalysts, into gold-containing zwitterionic intermediates that undergo different types of cycloaddition reactions with alkynes, alkenes or other unsaturated groups present in the reaction media. The cycloaddition usually generates a metal carbene that evolves through different pathways depending on the catalyst and on the reaction components. The reaction therefore can provide a variety of interesting polycyclic systems.

**Scheme 1:**
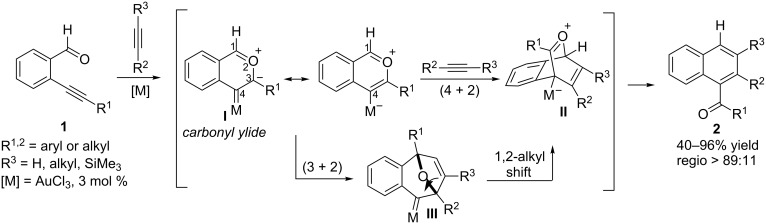
AuCl_3_-catalyzed benzannulations reported by Yamamoto.

For example, Yamamoto reported in 2002 a AuCl_3_-catalyzed formal (4 + 2) [23] benzannulation between *ortho*-alkynylbenzaldehydes of type **1** and alkynes (Scheme 1) [24–25]. The mechanism proposed by the authors involves an initial *5-endo* nucleophilic attack of the carbonyl moiety on the metal–alkyne complex to generate a carbonyl ylide intermediate **I**, which undergoes a regioselective (4 + 2) cycloaddition with the external alkyne. A subsequent elimination process on the resulting intermediate **II** accounts for the formation of the naphthyl ketone products **2**, which were isolated in good yields. More recently, in 2004, Straub and coworkers reported a DFT study on these cycloadditions that led them to propose a modification of the aforementioned mechanistic pathway [26]. According to the theoretical data, the formal (4 + 2) cycloaddition would indeed comprise a two-step process consisting of a dipolar (3 + 2) cycloaddition of the carbonyl ylide **I** to afford a carbene species **III** [27–28], followed by a 1,2-alkyl migration to yield the previously suggested intermediate **II** (Scheme 1).

The synthetic utility of these cycloadditions has also been explored, and recently an intramolecular version was applied to the synthesis of the angucyclinone antibiotics, (+)-ochromycinone and (+)-rubiginone B_2_ [29–32].

Closely related dipolar cycloadditions involving zwitterionic intermediates similar to **I** have also been developed with other transition metal catalysts such as tungsten, rhodium or platinum [32–35]. In particular, important developments were recently achieved with platinum catalysts [36–37], including the first enantioselective examples of these type of cycloadditions promoted by a chiral cationic platinum–diphosphine catalyst [38].

**Scheme 2:**
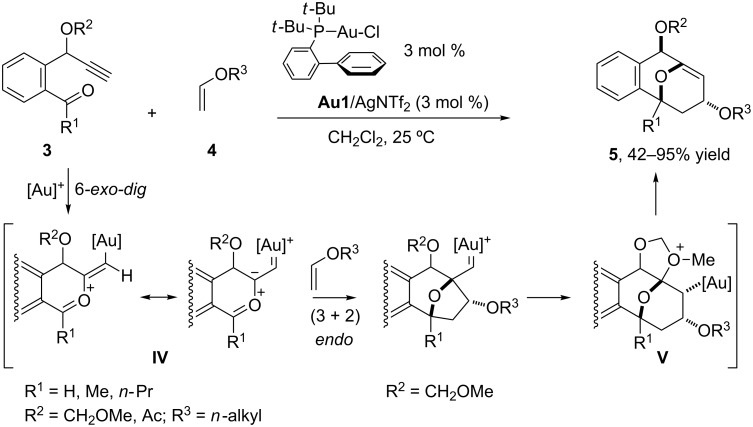
Synthesis of 9-oxabicyclo[3.3.1]nona-4,7-dienes from 1-oxo-4-oxy-5-ynes [40].

In contrast to these examples that proceed through an initial *endo-dig* cyclization and generate gold–carbonyl ylide species, the group of Liu recently demonstrated that it is also possible to produce alternative, reactive, zwitterionic intermediates of type **IV** through an *exo-dig* cyclization process when using 1-oxo-5-ynes such as **3** and a cationic gold complex prepared in situ from P(*t*-Bu)_2_(*o*-biphenyl)AuCl (**Au1**) [39] and AgNTf_2_ [40]. These dipolar intermediates undergo a cycloaddition with an external vinyl ether **4**, leading to interesting 9-oxabicyclo[3.3.1]nona-4,7-dienes **5** in good yields (Scheme 2). In view of the highly stereoselective outcome of these reactions and the requirement of an alkoxy or acyloxy group at the propargylic position of **3**, the authors proposed a reaction pathway based on an initial (3 + 2) dipolar cycloaddition between the carbonyl ylide **IV** and the alkene, followed by a ring expansion (1,2-alkyl migration) that is assisted by the oxy group and generates the oxonium intermediate **V** (Scheme 2). A final elimination process regenerates the catalyst and affords the oxacyclic product **5**. Importantly, the reaction also proceeds with non-aromatic 1-oxo-4-alkoxy-5-ynes [40].

**Scheme 3:**
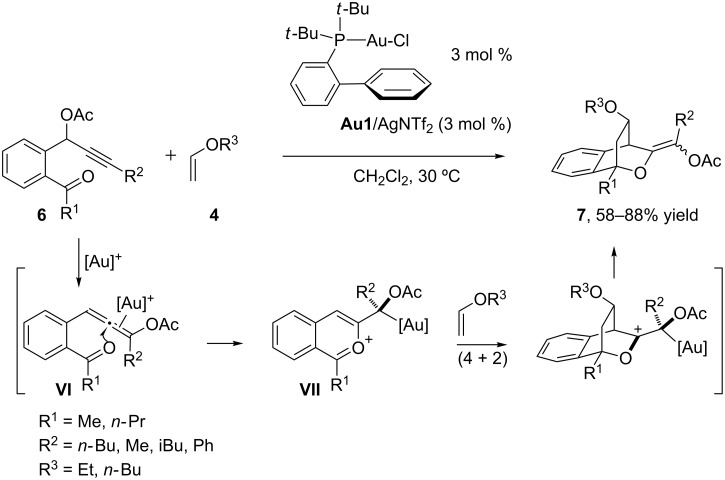
Stereocontrolled oxacyclization/(4 + 2)-cycloaddition cascade of ketone–allene substrates [43].

Curiously, when the substrate features an internal alkyne, such as in alkynyl acetate **6**, the reaction evolves through alternative mechanistic pathways [41]. In particular, Liu showed that in these cases, the gold activation of the alkyne promotes a 1,3-acyloxy shift that leads to ketone allenic intermediates of type **VI** (Scheme 3) [42–43]. Then, an intramolecular attack of the carbonyl group on the activated allene generates a benzopyrilium intermediate (**VII**) which undergoes a concerted and highly stereoselective (4 + 2) cycloaddition with the vinyl ether to yield, after the elimination of the gold catalyst, highly substituted oxacyclic systems **7** in good yields and with notable diastereoselectivities. Importantly, this reaction tolerates a wide range of vinyl ethers, and different substituents at the ketone and alkyne units, as well as a variety of substituents at the aromatic ring of **6**.

During the last few years, the groups of J. Zhang and L. Zhang have independently described alternative procedures to transform substrates that incorporate carbonyl and alkyne units into polar intermediates that undergo subsequent intermolecular annulations with diverse partners.

**Scheme 4:**
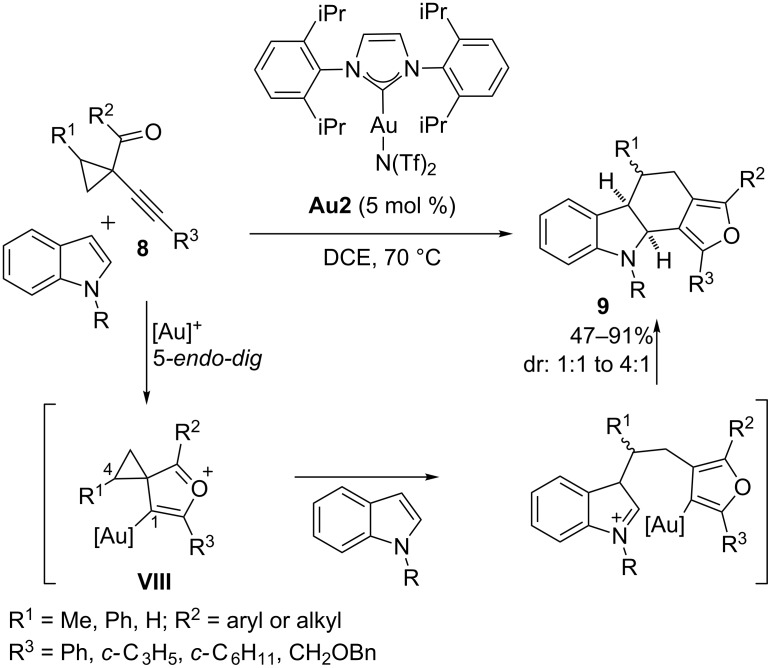
Gold-catalyzed synthesis of polycyclic, fully substituted furans from 1-(1-alkynyl)cyclopropyl ketones [45].

For example, in 2008, L. Zhang and coworkers demonstrated that a series of 1-(1-alkynyl)cyclopropyl ketones **8**, previously used by Schmalz for the synthesis of furans [44], can be used as precursors of reactive intermediates (**VIII**) that participate in (4 + 2) annulations with polarized alkenes such as indoles, carbonyls, imines or silyl enol ethers [45]. Thus, different types of 6-membered carbocycles and heterocycles were prepared in good yields and notable regioselectivities. An example of these annulations, using indoles as two carbon cycloaddition partners and IPrAuNTf_2_ as catalyst [**Au2**, IPr = bis(2,6-diisopropylphenyl)], is shown in Scheme 4. The reaction is initiated by a *5-endo-dig* cyclization of the carbonyl oxygen onto the Au-activated C–C triple bond, giving rise to the oxocarbenium intermediate **VIII**. The authors proposed that these intermediates formally behave as an all-carbon 1,4-dipole that intermolecularly reacts with the indole providing the final polycyclic furan adducts **9** in a regioselective fashion (Scheme 4) [46].

**Scheme 5:**
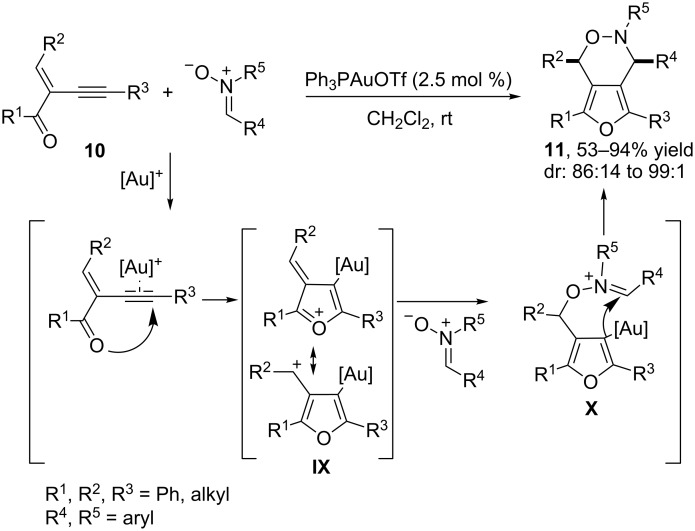
Gold-catalyzed 1,3-dipolar cycloaddition of 2-(1-alkynyl)-2-alken-1-ones with nitrones [47].

**Scheme 6:**
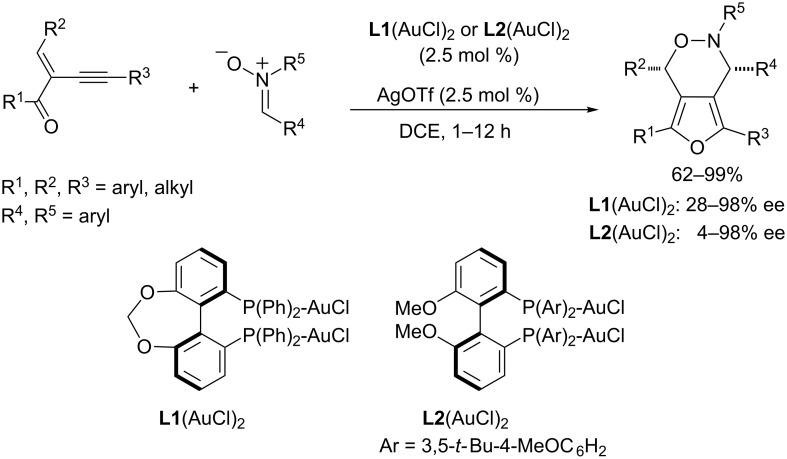
Enantioselective 1,3-dipolar cycloaddition of 2-(1-alkynyl)-2-alken-1-ones with nitrones [48].

In 2009, J. Zhang reported a gold(I)-catalyzed tandem cyclization/(3 + 3) cycloaddition of related 2-(1-alkynyl)-2-alken-1-ones **10** with nitrones [47]. The reactions provided a very practical entry to bicyclic oxazine derivatives **11**, which were obtained in good yields and excellent selectivities (Scheme 5). A plausible mechanism, proposed by the authors, consists of an initial activation of the alkyne group of **10** by the carbophilic gold catalyst (Ph_3_PAuCl/AgOTf), followed by an intramolecular cyclization that generates the furanyl–gold complex **IX**. This intermediate is then trapped by the nucleophilic oxygen atom of the nitrone to afford **X**, which eventually evolves to the final cycloadduct by means of an intramolecular cyclization reaction, which generally proceeds with diastereoselectivities higher than 95:5 (Scheme 5). Interestingly, these cycloadditions can also be carried out in a highly enantioselective fashion using any of the bis(gold) complexes derived from (*R*)-C_1_-tunephos [**L1**(AuCl)_2_] or (*R*)-MeO-dtbm-biphep [**L2**(AuCl)_2_], with the former being slightly more efficient in certain cases (Scheme 6) [48].

**Scheme 7:**
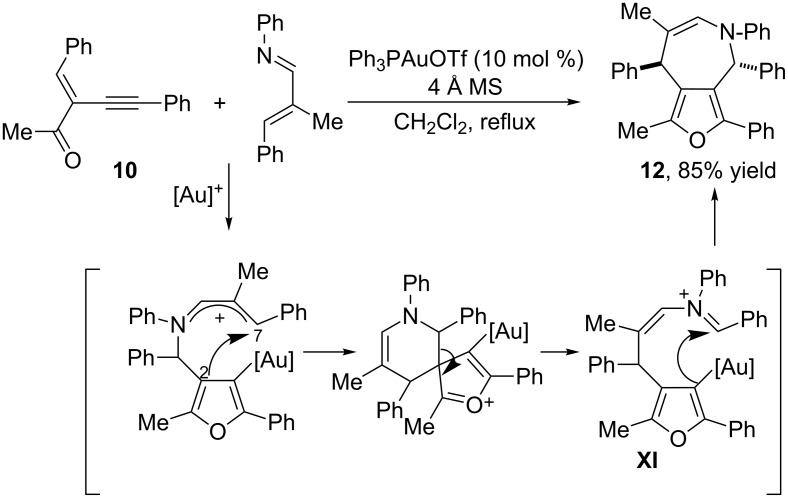
Gold-catalyzed 1,3-dipolar cycloaddition of 2-(1-alkynyl)-2-alken-1-ones with α,β-unsaturated imines [49].

If instead of a nitrone, a nucleophilic α,β-unsaturated imine is used as the second cycloaddition component, furo[3,4-*c*]azepines such as **12** can be obtained [49]. An example of these cycloadditions is shown in Scheme 7. The mechanistic pathway proposed by the authors is also based on the interception of the furanyl–gold complex **IX** (see Scheme 5) by the nucleophile (the unsaturated imine), which is now followed by a 2,7-cyclization process and a ring cleavage to yield a furanyl intermediate featuring an iminium ion (**XI**). This species undergoes an intramolecular cyclization to yield the observed azepine product, and regenerates the gold catalyst.

**Scheme 8:**
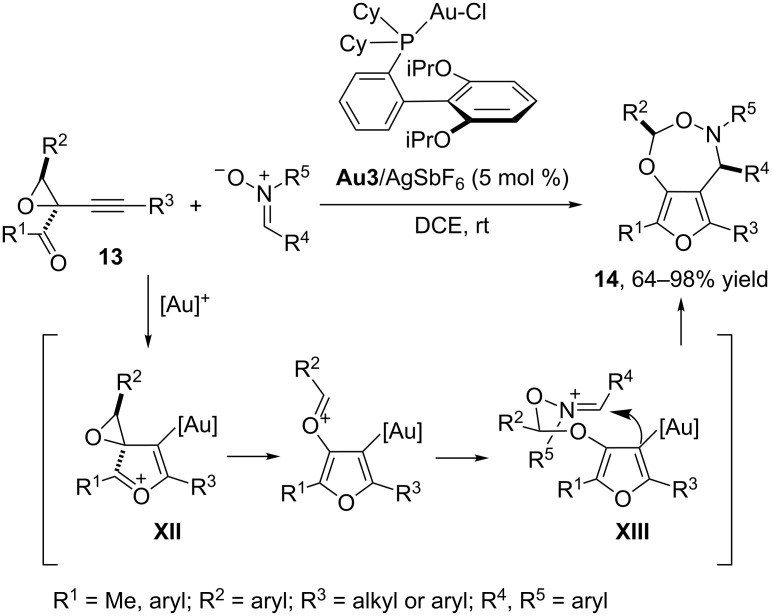
Gold-catalyzed (4 + 3) cycloadditions of 1-(1-alkynyl)oxiranyl ketones [50].

Very recently, the same group reported a related gold-triggered formal (4 + 3) cycloaddition between nitrones and 1-(1-alkynyl)oxiranyl ketones **13** [50]. The method provides heterobicyclic products of type **14** in a highly diastereoselective fashion. In these reactions, the gold complex **Au3**, derived from the bulky biaryl phosphine ligand RuPhos, provided the best reaction yields (Scheme 8). From a mechanistic point of view, the authors proposed an initial nucleophilic attack of the carbonyl oxygen on the gold(I)-activated alkyne to form a vinyl–gold intermediate **XII**, analogous to that previously shown in Scheme 5 (**IX**). Aromatization of this intermediate through C–C bond cleavage of the oxirane unit, followed by addition of the nitrone, delivers intermediate **XIII**, which evolves to the final cycloadduct by ring closure through a favored chair-like conformation. An attack of the nitrone on intermediate **XII**, to directly generate species **XIII**, has also been proposed as an alternative pathway.

**Scheme 9:**
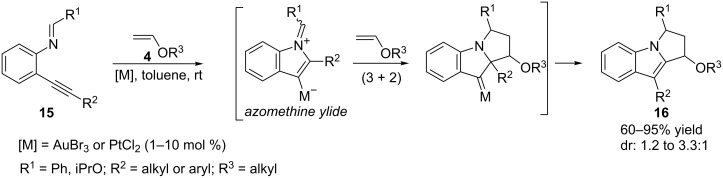
(3 + 2) Cycloaddition of gold-containing azomethine ylides [52].

Common to all these reported cycloadditions is the initial nucleophilic addition of a carbonyl oxygen to the alkyne. As expected, an imine can also be used as a nucleophile, such as **15**, which leads to the generation of an azomethine ylide capable of participating in dipolar (3 + 2) cycloadditions to unsaturated systems such as electron-rich alkenes. Examples of these cycloadditions, originally reported under tungsten catalysis [51], have been recently reported by Iwasawa with Au(III) and Pt(II) catalysts [52], allowing the assembly of interesting tricyclic indole skeletons **16** in good yields (Scheme 9).

**Scheme 10:**
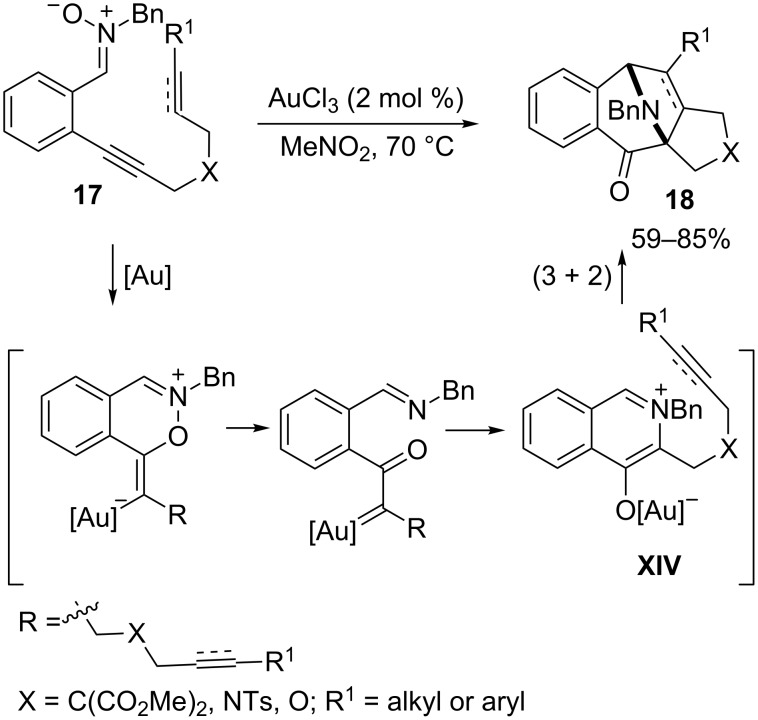
Gold-catalyzed generation and reaction of azomethine ylides [53].

In 2008, Shin and coworkers reported an alternative procedure for the generation and subsequent cycloaddition of azomethine ylide intermediates under gold catalysis. Importantly, they demonstrated that the intramolecular attack of a nitrone oxygen to a tethered gold-activated alkyne leads, by means of an internal redox reaction, to an α-carbonyl carbenoid tethered to an imine group (Scheme 10). A subsequent attack of this imine to the carbenoid generates the reactive azomethine ylide intermediate **XIV**, which undergoes a (3 + 2) dipolar cycloaddition with an intramolecularly tethered alkene or alkyne. Thus, interesting azabicyclo[3.2.1]octane skeletons **18** could be obtained in a highly diastereoselective fashion, in good yields and in excellent atom-economy [53–54].

**Scheme 11:**
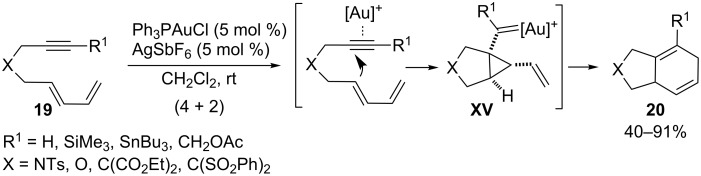
Gold-catalyzed intramolecular (4 + 2) cycloadditions of unactivated alkynes and dienes [55].

The above reactions involve an initial attack of a heteroatom on gold-activated alkynes. However, it is also possible to induce alternative cycloaddition reactions of alkynes that start by addition of a π-carbon nucleophile instead of a heteronucleophile. For instance it has been shown that it is possible to achieve gold-catalyzed formal intramolecular (4 + 2) cycloadditions between alkynes and non-activated dienes such as **19** (e.g., Scheme 11) [55]. This type of cycloaddition has been classically promoted by other metal complexes such as Rh [56], Ru [57], or Ni [58], among others, that are metals that usually promote reaction pathways via metallacyclic intermediates in which the metal atom undergoes redox changes. In the case of the gold-catalyzed process the mechanism does not involve a change in the oxidation level of gold. Indeed, it has been proposed that the alkyne activation promotes a cyclization that generates a key cyclopropyl carbene intermediate of type **XV**. This intermediate evolves to the final cycloadduct **20** through a rearrangement process in which the gold catalyst is regenerated.

**Scheme 12:**
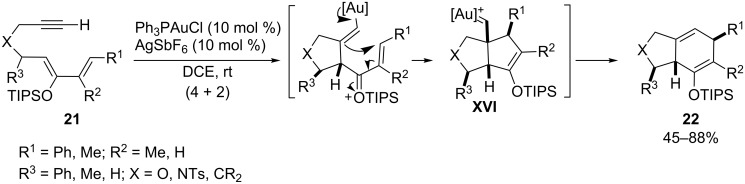
Gold-catalyzed preparation of bicyclo[4.3.0]nonane derivatives from dienol silyl ethers [59].

When using a dienol silyl ether such as **21** (Scheme 12) as the diene component, the formation of the (4 + 2) products can be justified in terms of an alternative mechanism consisting of a 5-exo nucleophilic attack of the silyl enol ether moiety on the electrophilically activated alkyne, followed by addition of the generated alkenyl metallic species to the α,β-unsaturated silyl oxonium moiety, to give a bicyclic carbene intermediate **XVI** [59]. These species, which do not incorporate an α-hydrogen atom that could participate in a 1,2-hydrogen shift process, evolve through a 1,2-alkyl migration to give the ring-expanded products **22**, formally (4 + 2) cycloadducts. Interestingly, the stereoselectivity of these products is different from that of the thermal Diels–Alder adducts that result when the substrates are heated under reflux in toluene.

**Scheme 13:**
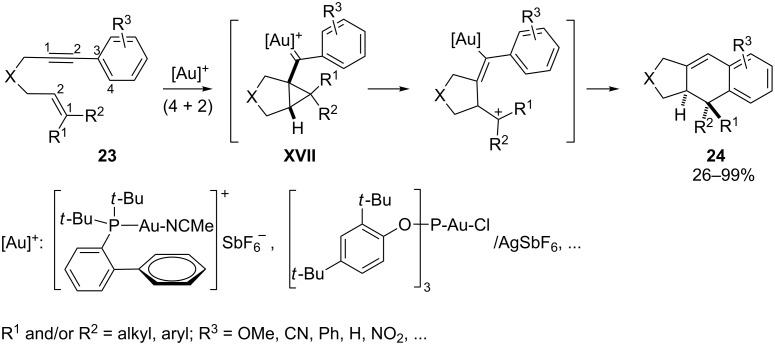
Gold(I)-catalyzed intramolecular (4 + 2) cycloadditions of arylalkynes or 1,3-enynes with alkenes [60].

The use of 1-aryl-1,6-enynes such as **23** and cationic Au(I) catalysts also allows one to perform a complementary type of formal (4 + 2) cycloaddition reaction (Scheme 13). In these reactions, developed by Echavarren and coworkers, the intermediate carbene species of type **XVII** evolves through ring-opening of the cyclopropanic unit followed by a Friedel–Crafts-type cyclization, which completes the catalytic cycle and regenerates the gold catalyst [39,60].

**Scheme 14:**
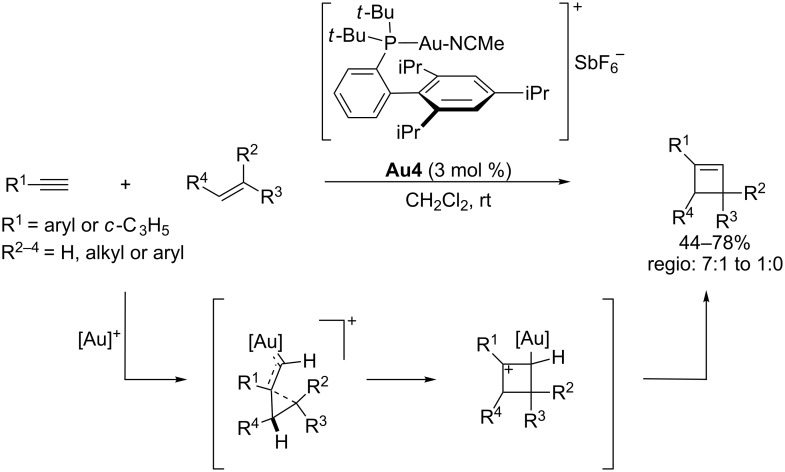
Gold(I)-catalyzed intermolecular (2 + 2) cycloaddition of alkynes with alkenes [62].

Interestingly, small changes in the substitution of the alkene, or the use of other catalysts, such as PtCl_2_ (under a CO atmosphere), affect the result of the annulation, such that it is now possible to induce (2 + 2) instead of (4 + 2) cycloadditions [61]. Very recently, Echavarren and coworkers reported an intermolecular variant of this type of (2 + 2) alkyne–alkene cycloaddition reaction (Scheme 14) [62–63].

**Scheme 15:**
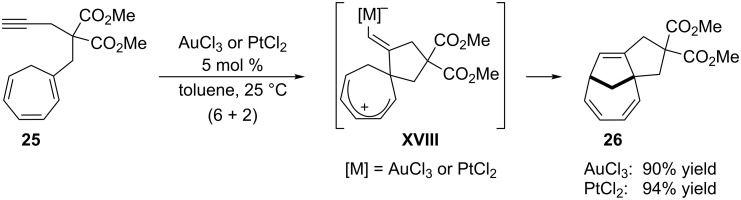
Metal-catalyzed cycloaddition of alkynes tethered to cycloheptatriene [65].

A less common annulation is the intramolecular (6 + 2) cycloaddition between non-activated alkynes and a cycloheptatriene. These cycloadditions were previously reported in the context of a stoichiometric chromium(0) activation of the triene unit [64]. The use of AuCl_3_ or PtCl_2_ rendered the reaction catalytic [65]. The mechanism entails a stepwise *exo*-cyclization of the cycloheptatriene onto the gold-activated alkyne, and ring closure of the resulting pentadienyl cation species (**XVIII**) to give the final tricyclic adducts in good yields. Although PtCl_2_ is the most efficient catalyst for these intramolecular cycloadditions, the reaction of substrate **25** can also be performed with 5 mol % of AuCl_3_ at room temperature, resulting in good yield (Scheme 15).

**Scheme 16:**
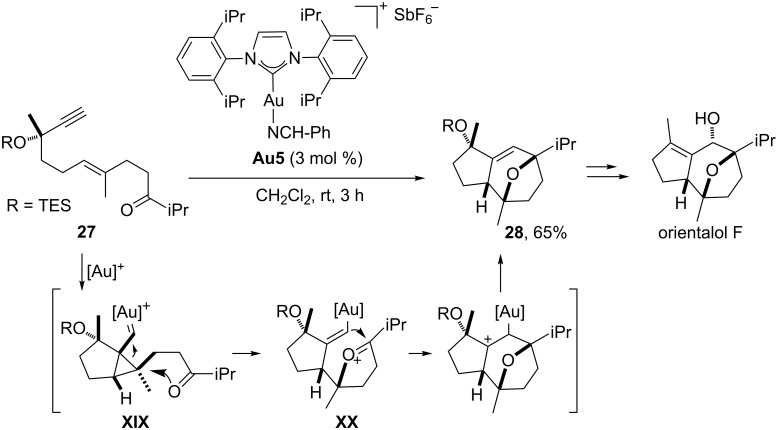
Gold-catalyzed cycloaddition of functionalized ketoenynes: Synthesis of (+)-orientalol F [68].

In addition to all of these cycloadditions involving the participation of an alkyne and a second component, several gold-catalyzed formal cycloadditions of three different reaction components have also been described. In particular, the group of Echavarren has recently developed a formal (2 + 2 + 2) gold-catalyzed synthesis of interesting oxa-bridged bicyclo[5.3.0]decanes from 1,6-enyne precursors equipped with an appropriately tethered carbonyl group (**27**, Scheme 16) [66]. In these processes, the carbonyl acts as a nucleophile, attacking the gold–cyclopropyl carbene intermediate **XIX** to generate an oxonium cation species of type **XX**. Finally, a Prins-like cyclization renders the oxatricyclic product **28** and regenerates the gold(I) catalyst. Importantly, this strategy was successfully applied as a key step in the synthesis of orientalol F (Scheme 16) and englerins A and B [67–69].

**Scheme 17:**
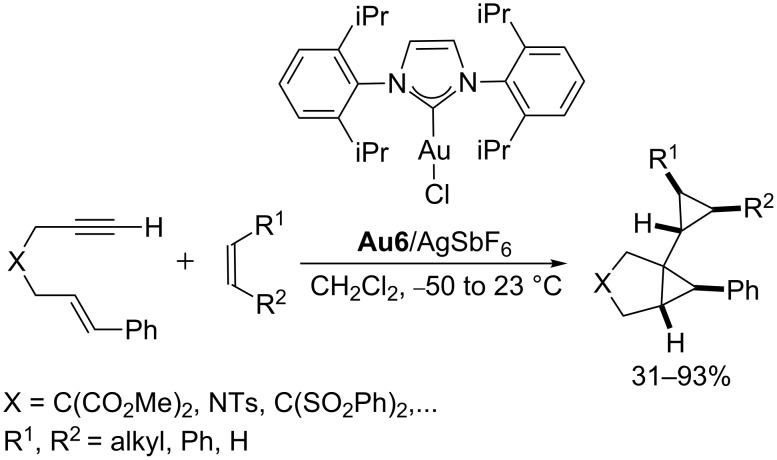
Gold-catalyzed intermolecular cyclopropanation of enynes with alkenes [70].

Alternatively, the use of an alkene instead of a carbonyl nucleophile allows one to trap the carbene gold(I) intermediate in a (2 + 1) cycloaddition process that renders dicyclopropyl products. Both, intra- and intermolecular variants of these reactions have been reported in recent years (Scheme 17) [70–71].

**Scheme 18:**
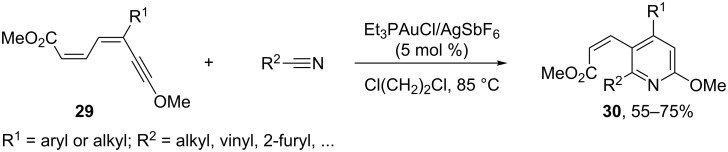
Gold-catalyzed intermolecular hetero-dehydro Diels–Alder cycloaddition [72].

Finally, the activation of alkynes with Au(I) has also been used recently to induce a hetero-dehydro Diels–Alder type of reaction. In particular, certain dienynes **29** with alkoxy groups at position 1 undergo a (4 + 2) cycloaddition with nitriles in the presence of cationic gold catalysts, such as Et_3_PAuCl/AgSbF_6_, to give tetrasubstituted pyridines **30** in good yields (Scheme 18) [72].

### Cycloadditions initiated by gold-activation of propargyl esters

These cycloadditions are a special case of those based on the activation of alkynes and deserve a separate discussion due to their relevance, wide versatility, and mechanistic peculiarities.

**Figure 1:**
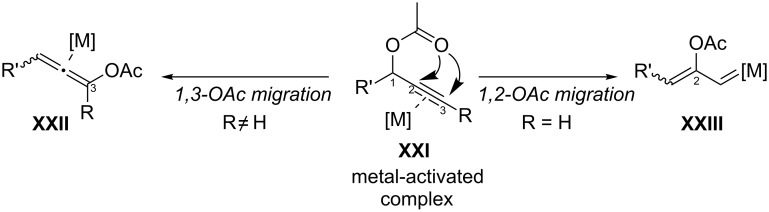
Gold-catalyzed 1,2- or 1,3-acyloxy migrations of propargyl esters.

Propargyl esters, usually acetates, are prone to undergo 1,2- or 1,3-acyloxy migrations in the presence of gold catalysts. The migration process begins with the nucleophilic intramolecular attack of the carboxyl unit on the metal-activated alkyne complex **XXI**. When a terminal alkyne is used (Figure 1, R = H), the 1,2-migration of the acetate moiety is preferred, affording an alkenyl–gold carbenoid species of type **XXIII**. In contrast, internal alkynes typically experience a 1,3-acyloxy migration rendering allenyl acetates of type **XXII** (Figure 1, R ≠ H), species which can be additionally activated by the metal catalyst to afford a wide range of gold-catalyzed transformations. Theoretical studies showed that all these species are in rapid equilibrium and the reactivity of the system depends not only on the substrate but also on the particular type of gold catalyst that is employed [73].

**Scheme 19:**
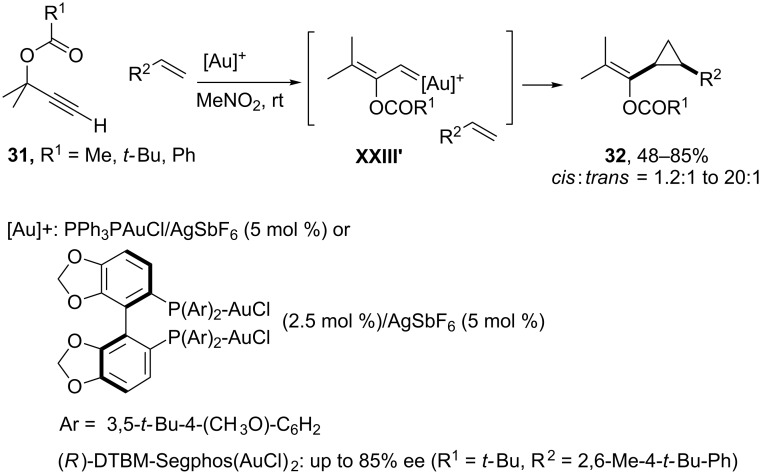
Gold(I)-catalyzed stereoselective olefin cyclopropanation [74].

Several groups, in particular the group of Toste, have exploited the chemistry of these systems to develop new types of cycloaddition reactions [74–76]. For example, they showed that it is possible to trap the intermediate gold carbenoids of type **XXIII’**, resulting from 1,2-acyloxy migration on propargyl esters such as **31** (pivalates, acetates or benzoates), with external alkenes. Usually, the reactions are highly stereoselective, predominantly affording the *cis*-cyclopropanic adduct **32**. Moreover, the reaction tolerates a wide range of olefin substitutions (from mono- to tetra-substituted alkenes) and can be performed, in some cases, with excellent enantioselectivity using bis(gold) complexes derived from DTBM-Segphos (Scheme 19) [74–76].

**Scheme 20:**
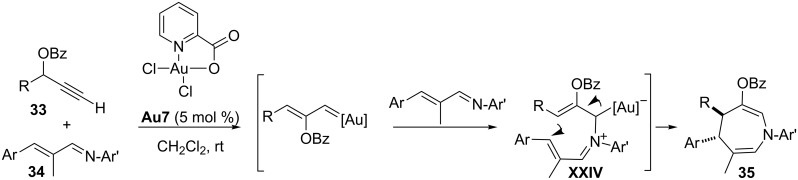
Reaction of propargylic benzoates with α,β-unsaturated imines to give azepine cycloadducts [77].

Additionally, Toste and coworkers also described the reaction of propargylic benzoates with α,β-unsaturated imines to give azepine products **35** with excellent yields [77]. The formal (4 + 3) cycloaddition takes place in three basic stages: 1) Generation of the gold carbenoid by a 1,2-acyloxy migration, 2) attack of the imine on these electrophilic species to give an allyl–gold intermediate **XXIV**, and 3) final intramolecular cyclization to give the seven-membered heterocycle, a process that occurs with high diastereoselectivity. Critical for the success of this reaction is the use of the picolinic acid-derived gold(III) catalyst **Au7** (Scheme 20).

**Scheme 21:**
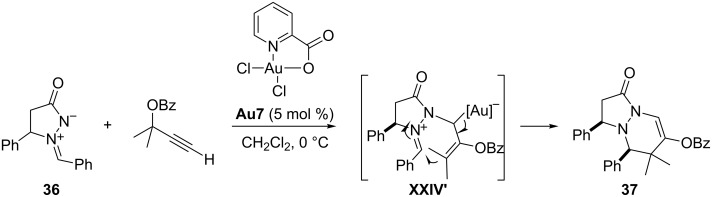
Gold-catalyzed (3 + 3) annulation of azomethine imines with propargyl esters [81].

This new gold-catalyzed transformation is somewhat related to those previously described by Doyle and by Barluenga that involved α,β-unsaturated imines and rhodium–vinyl carbenoids (Doyle) or Fischer carbenes (Barluenga). However, the stereochemical outcome of these three processes is different [78–79]. Therefore, the reactivity of gold species of type **XXIII** may be sometimes similar to that of other transition metal carbenoids or even to that of alkenyl Fischer carbenes. However, in many other cases, it is markedly different. For instance, while alkenyl Fischer carbenes act as two-carbon atom components in (3 + 2) cycloadditions with 1,3-dipoles [80], gold–carbenoids of type **XXIII**, generated from propargyl esters, can work as three-carbon synthons in cycloadditions with azomethine imines, also reported by Toste. An example is shown in Scheme 21 [81]. These (3 + 3) annulations take place through a stepwise mechanism related to that for the formation of azepines, consisting of a nucleophilic attack of the azomethine imines onto the intermediate alkenyl–gold carbenoid to afford an allyl–gold species **XXIV’**, which evolves to the final adduct through a stereoselective ring closure (Scheme 21). The scope of the method is rather broad, as it tolerates tertiary and secondary propargyl esters as well as the presence of several different substituents at the azomethine imine component. The picolinic acid-derived gold(III) catalyst **Au7** provided the best results.

**Scheme 22:**
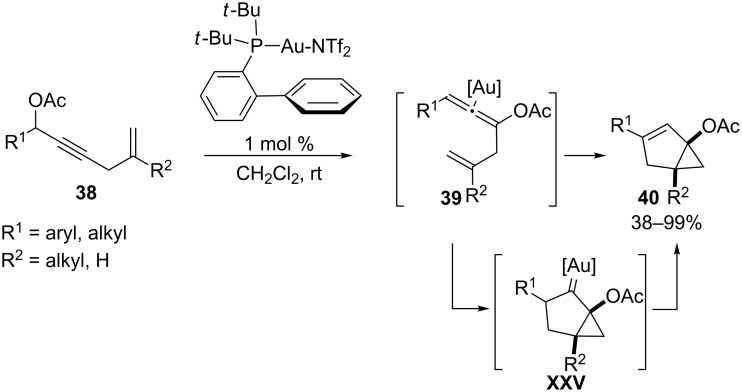
Gold(I)-catalyzed isomerization of 5-en-2-yn-1-yl acetates [83].

On the other hand, allenyl acetates of type **XXII**, resulting from a 1,3-acyloxy migration of propargyl acetates can also participate in myriad gold-catalyzed cycloaddition reactions [82]. In 2006, Gagosz and co-workers reported a gold(I)-catalyzed isomerization of enynyl acetates such as **38** to afford bicyclo[3.1.0]hexenes **40** with excellent yields and stereoselectivities [83]. The authors demonstrated that these reactions are initiated by the 1,3-migration of the ester group to provide an allenyl ester intermediate **39**, which could be isolated and further transformed into the final products under the same catalytic conditions [84]. Thus, the authors proposed that the gold(I) catalyst is able to activate these allenic intermediates in situ*,* triggering a stepwise intramolecular (3 + 2) annulation reaction with the pendant alkene. This cycloaddition provides a cyclic gold carbene species **XXV**, which is eventually transformed into the final bicyclic adduct by a 1,2-hydrogen shift, with concomitant regeneration of the gold(I) catalyst (Scheme 22). Although the authors did not catalogue this method as a cycloaddition reaction, it can be considered as a pioneering example of a formal gold-catalyzed (3 + 2) intramolecular cycloaddition reaction, occurring in a stepwise fashion.

**Scheme 23:**
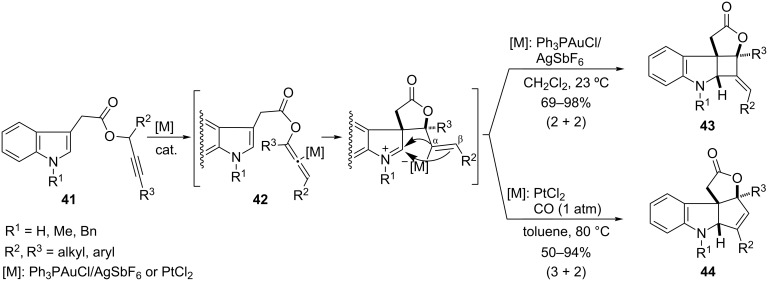
(3 + 2) and (2 + 2) cycloadditions of indole-3-acetates **41** [85–86].

The group of L. Zhang also developed several annulation reactions of allenyl esters generated in situ by a metal-catalyzed 1,3-migration of propargyl precursors [85–86]. In particular, they showed that propargyl indole-3-acetates **41** undergo gold(I)- or platinum(II)-catalyzed 1,3-migration to acyloxy allenic esters **42**; compounds which evolve in situ in the presence of the same metal catalyst to give adducts resulting from formal (3 + 2) and/or (2 + 2) annulation processes (Scheme 23). In the presence of Ph_3_PAuCl/AgSbF_6_, the (2 + 2) cycloaddition is favored furnishing **43** (upper arrow) [85]. However, when PtCl_2_ (under an atmosphere of CO) is used, the major products are 2,3-indoline-fused cyclopentenes **44**, which arise from a formal (3 + 2) cycloaddition (lower arrow) [86]. Thus, the appropriate choice of a Pt or Au catalyst determines whether the allenyl intermediate **42** participates as a 2C- or as a 3C-atom component in the annulation. A precise explanation for this dichotomy has not been specifically addressed.

### Cycloadditions initiated by gold-activation of allenes

**Scheme 24:**
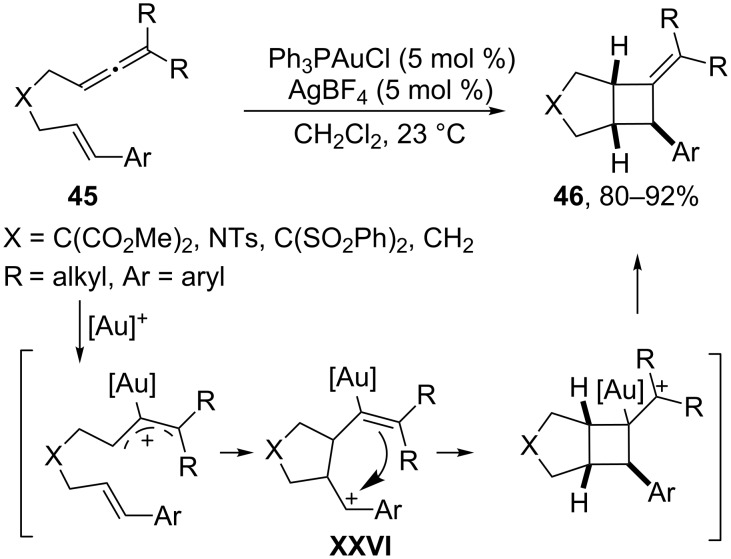
Gold(I)-catalyzed (2 + 2) cycloaddition of allenenes [87].

Several gold catalysts can activate allenes in a very chemoselective way, triggering different types of cycloaddition processes. We have seen in the previous section some examples of cycloadditions involving allenes, in particular with acyloxy allenes, generated in situ by activation of propargyl esters with gold and/or platinum catalysts. It is therefore reasonable to assume that other allenes can also participate in these or related cycloaddition reactions. Indeed, the group of Toste described in 2007 a (2 + 2) intramolecular cycloaddition reaction between allenes and alkenes by gold catalysis [87]. The proposed mechanism is based on an activation of the allene to give a cationic metal species which undergoes a cyclization to give a stabilized carbocation **XXVI**, usually a benzylic cation (Scheme 24). A subsequent ring closure through the carbon adjacent to the gold atom provides the final cycloadduct **46**, featuring a four membered ring carbocycle. Importantly, these reactions could be rendered enantioselective with catalysts derived from DTBM-Segphos or, as recently demonstrated, with gold complexes derived from chiral phosphoramidites [88–89].

**Scheme 25:**
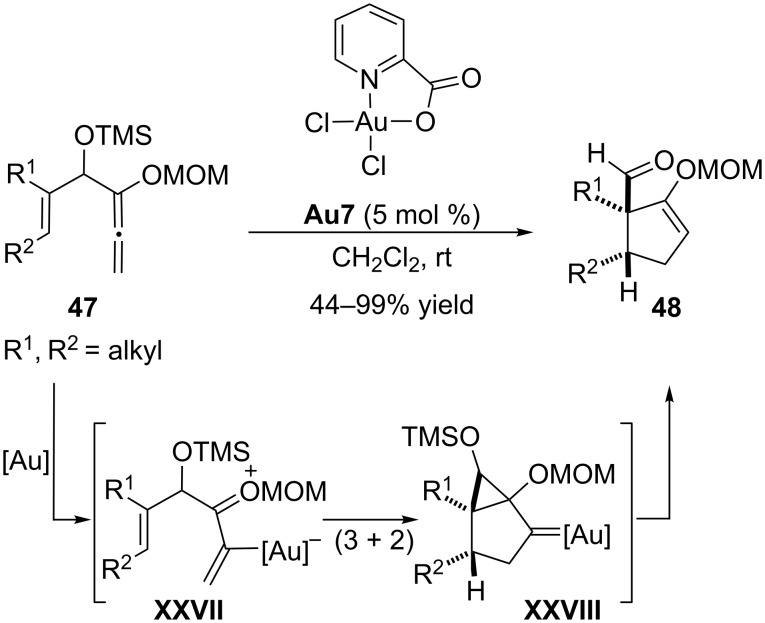
Formal (3 + 2) cycloaddition of allenyl MOM ethers and alkenes [90].

Also in 2007, L. Zhang and co-workers reported another formal intramolecular cycloaddition between alkenes and allenes, in particular a (3 + 2) cycloaddition between allenyl MOM ethers and alkenes (MOM = methoxymethyl, Scheme 25) [90]. The activation of these allenes by the dichloro(pyridine-2-carboxylato)Au(III) complex **Au7** generates an oxocarbenium intermediate **XXVII**, which undergoes the (3 + 2) annulation with the alkene. The resulting bicyclo[3.1.0] species **XXVIII**, related to those previously proposed by Gagosz, evolves through a cyclopropane fragmentation and protodeauration to afford the products **48** in good yields and excellent stereoselectivities.

**Scheme 26:**
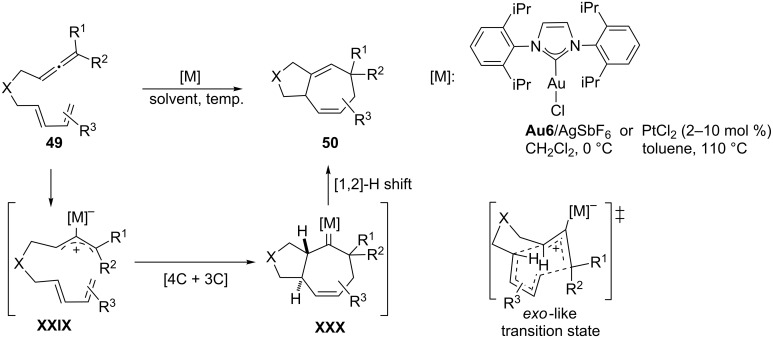
(4 + 3) Cycloadditions of allenedienes [97–98].

Based on these and other reports demonstrating the ability of gold(I) and platinum(II) catalysts to induce reactions of allenes through cationic intermediates [91–94], we investigated the possibility of using allenes as allyl cation surrogates, such that they could participate in concerted [4C(4π) + 3C(2π)] cycloadditions with conjugated dienes, a similar process to those previously reported between oxyallyl cations and dienes [95–96]. Initially, we found that PtCl_2_ was an excellent catalyst for promoting these intramolecular [4C + 3C] cycloadditions between 1,3-dienes and allenes (Scheme 26) [97]. DFT calculations as well as experimental data agreed with a mechanistic pathway based on the metal activation of the allene to afford a metal–allyl cation intermediate of type **XXIX**, which subsequently undergoes a concerted (4 + 3) cycloaddition reaction with the diene. The resulting metal carbene species (**XXX**) eventually evolves through a 1,2-hydrogen shift, leading to seven-membered carbocycles **50** and regenerating the catalyst (PtCl_2_). In addition, DFT calculations suggested that gold catalysts could be even more active than PtCl_2_. Accordingly, the use of a cationic Au(I) catalyst containing a σ-donating *N-*heterocyclic carbene ligand (**Au6**/AgSbF_6_, Scheme 26) enabled these reactions at lower temperatures and increased the scope and synthetic utility of the process [98]. In general, the reactions are completely diastereoselective affording the products as a result of an *exo-*like approach of the allyl cation to the diene. The reaction tolerates a variety of substituents on the allene and the diene, providing a variety of bicyclo[5.3.0]decane systems in good yields.

**Scheme 27:**
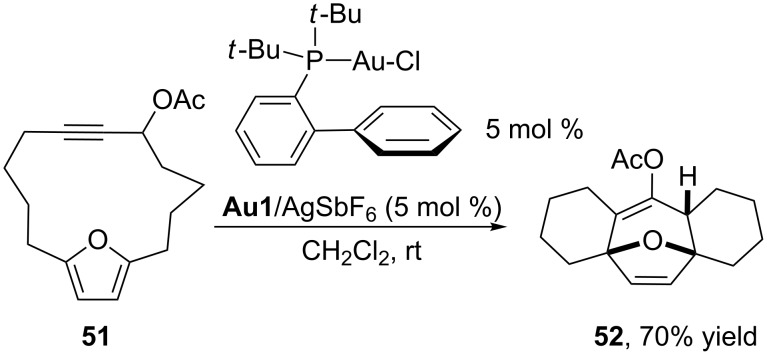
Gold-catalyzed transannular (4 + 3) cycloaddition reactions [101].

Other gold(I) complexes, such as that including a highly donating biaryl di-*tert*-butylphosphine ligand **Au1**, also allow these cycloadditions, as recently reported by Gung and by Toste [99–101]. In particular, an interesting transannular cycloaddition was performed on the substrate **51** equipped with a furan (4C) and a propargyl acetate, which formally acts as an allene surrogate (Scheme 27).

**Scheme 28:**
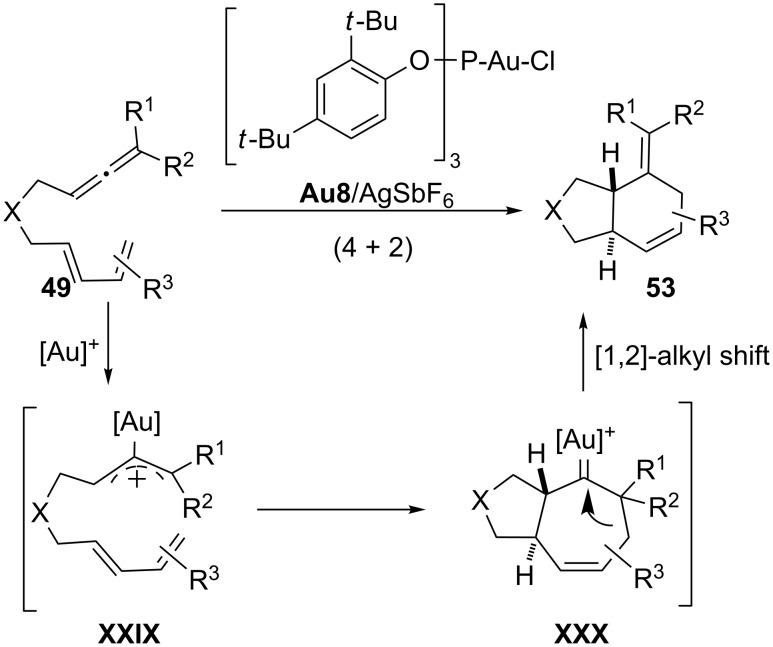
Gold(I)-catalyzed (4 + 2) cycloadditions of allenedienes [102].

Curiously, the allenedienes **49**, when disubstituted at the distal position of the allene, give rise to formal (4 + 2) cycloaddition products **53** when treated with a gold(I) catalyst bearing a π-acceptor ligand, such as a triarylphosphite (**Au8**/AgSbF_6_, Scheme 28) [98–99,102–103]. Several experimental results as well as theoretical calculations suggest that the observed (4 + 2) cycloadducts **53** are indeed the result of a ring contraction process (1,2-alkyl shift) in the initially formed cycloheptenyl–gold carbene intermediate **XXX** (Scheme 28). Thus, the ligand at gold determines the fate of this carbene and hence the formation of the (4 + 3) (**50**) or (4 + 2) (**53**) cycloadducts.

**Scheme 29:**
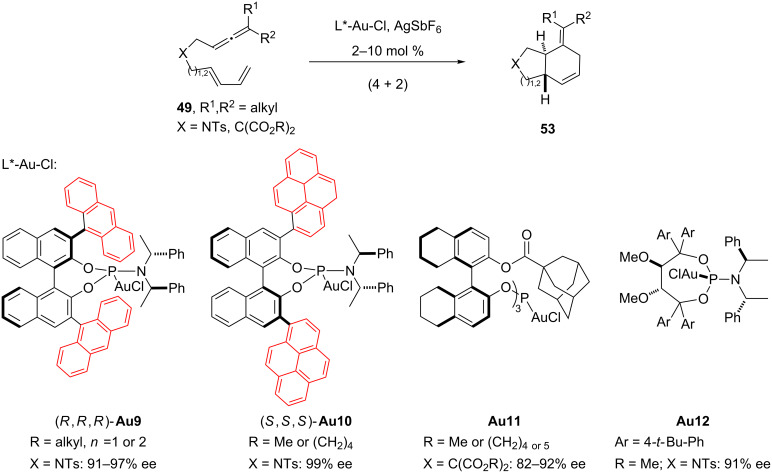
Enantioselective gold(I)-catalyzed (4 + 2) cycloadditions of allenedienes [88,102,104].

Based on the electronic similarity between phosphites and phosphoramidites, we also studied enantioselective variants of this cycloaddition using gold complexes derived from this highly versatile type of chiral ligand. We found that it was possible to obtain excellent enantioselectivities with gold complexes derived from bulky phosphoramidites with anthracenyl substituents at 3 and 3' positions of the binaphthol moiety (**Au9**, Scheme 29) [102]. Almost simultaneously, the group of Toste reported that related phosphoramidite–gold complexes, such as **Au10,** and the chiral gold catalyst **Au11** [104], derived from a C_3_-symmetric phosphite ligand previously developed by Reetz and coworkers [105], are also capable of inducing excellent enantioselectivities in these (4 + 2) cycloaddition reactions of allenedienes. More recently, the group of Fürstner has also reported that Taddol-based phosphoramidite–gold complexes such as **Au12** (Scheme 29) are excellent catalysts for these (4 + 2) processes, as well as for the (2 + 2) cycloadditions of eneallenes such as those shown in Scheme 24 [88].

**Scheme 30:**
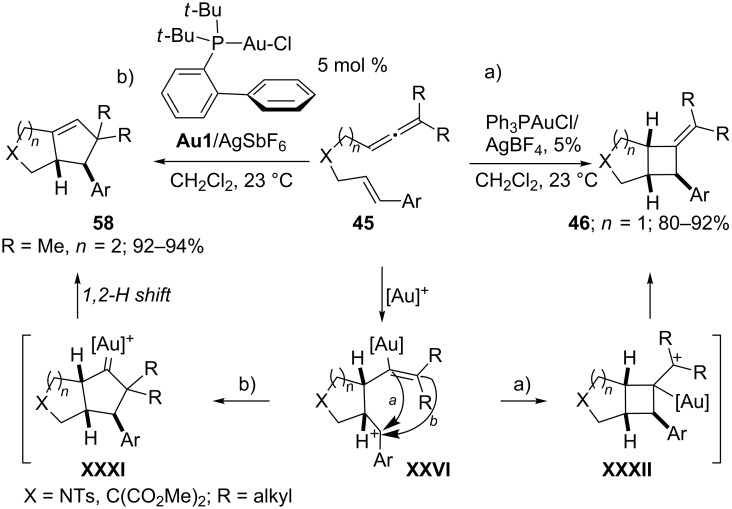
(3 + 2) versus (2 + 2) Cycloadditions of allenenes [87,99].

In addition to these allene–diene cycloadditions in which the type of gold catalyst determines whether the (4 + 2) or the (4 + 3) adduct is formed, Toste and Fürstner independently reported additional examples confirming that, depending on the ancillary ligands at gold, the allenes can behave either as 2C- or 3C-components in their intramolecular annulations to alkenes [99,106–107]. As previously shown in Scheme 24, the reaction of eneallene **45** with Ph_3_PAuCl/AgBF_4_ provides alkylidenecyclobutanes **46**, formally resulting from an internal (2 + 2) cycloaddition [87]. However, in 2009, Toste demonstrated that, when a gold catalyst such as **Au1**/AgSbF_6_, with a more readily donating phosphine ligand is employed, the alternative (3 + 2) cycloaddition leading to bicyclo[4.3.0]nonanes **58** is favored (Scheme 30) [99]. The authors propose stepwise mechanisms proceeding through a common cationic intermediate **XXVI**, which can evolve into the cyclopentyl cycloadducts via the species **XXXI**. Alternatively, when the benzylic carbocation in **XXVI** is intercepted by the carbon atom bearing the gold atom, the (2 + 2) adduct **46** is formed. Nonetheless, the formation of adducts **46** by a ring contraction process in intermediate **XXXI** cannot be fully discarded.

**Figure 2:**
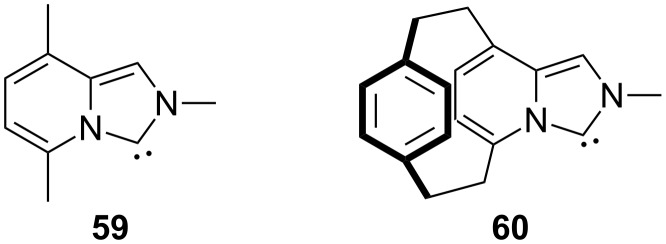
NHC ligands with different π-acceptor properties [106].

More recently, Fürstner and coworkers have further demonstrated this type of dichotomy depending on the electronic characteristics of the ligands at gold (Figure 2) [106]. In particular, they showed that the π-acceptor properties of NHC ligands such as **59** could be enhanced by introducing a second aromatic layer spanning the imidazopyridine-2-ylidene system, whereas the σ-donating abilities of both ligands remain basically equivalent.

**Scheme 31:**
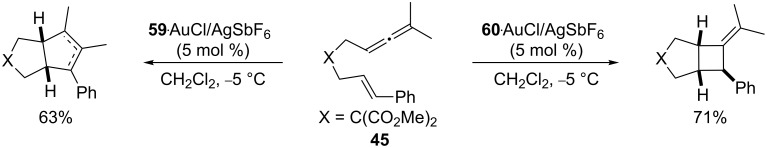
(3 + 2) versus (2 + 2) Cycloadditions of allenenes [106].

As a consequence, the new cyclophanic NHC ligand **60** and the related imidazopyridine-2-ylidene analog **59**, generate gold complexes that behave in a divergent manner. While complex **59**–AuCl, containing a poor π-acceptor ligand, is able to efficiently induce the (3 + 2) cycloaddition of eneallene **45** (R = Me, X = C(CO_2_Me)_2_, Ar = Ph, *n* = 1, Scheme 30), a related catalyst with the cyclophanic NHC ligand (**60**–AuCl) afforded the (2 + 2) cycloadduct with high selectivity and good yield (Scheme 31). From a mechanistic point of view, the authors suggested that both catalysts initially provide the common intermediate **XXVI** (Scheme 30). Then, a cationic catalyst derived from **59**–AuCl could favor the formation of the formal (3 + 2) cycloadduct via intermediate **XXXI**, that can also be interpreted as a gold-stabilized carbocationic species. On the contrary, a more electron-deficient catalyst such as **60**–AuCl/AgSbF_6_ could favor the formation of intermediate **XXXII**, in which a cationic center is not directly bound to the gold complex, therefore leading to the (2 + 2) adduct (Scheme 30 and Scheme 31).

Importantly, the same NHC–Au complexes were also able to selectively induce either the (4 + 3) or the (4 + 2) cycloadditions of allenedienes **49** (R^1^, R^2^ = Me, R^3^ = H, X = C(CO_2_Me)_2_). These examples clearly demonstrate the possibility of modulating the electronic properties of reactive intermediates generated upon activation of allenes with Au(I) complexes, and thereby influence the reaction outcome.

**Scheme 32:**
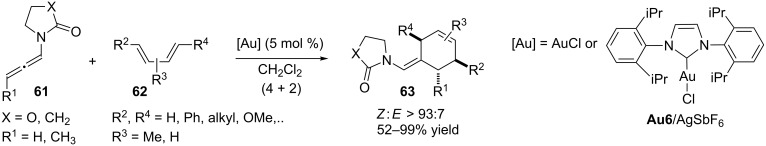
Gold(I)-catalyzed intermolecular (4 + 2) cycloaddition of allenamides and acyclic dienes [109].

The development of intermolecular variants of gold-catalyzed cycloadditions with allenes remains much less studied, probably due to the inherent difficulties in controlling not only the chemo- but also the regioselectivity of the process. The first examples of an intermolecular cycloaddition with allenes and carbophilic catalysts were reported in 2009 by Iwasawa and consisted of an intermolecular (3 + 2) cycloaddition between allenes and enol silyl ethers, catalyzed by a platinum(II) catalyst [108]. Only in 2011, were the first examples with gold catalysts reported. In particular, an intermolecular (4 + 2) cycloaddition was described by our group between allenamides **61** and conjugated dienes **62** (Scheme 32); a process that provided a straight entry to a variety of differently substituted cyclohexenes **63**, and took place with excellent regio- and diastereoselectivity [109]. Almost simultaneously a (4 + 2) cycloaddition between allenyl ethers and dienes was also reported by Goeke using Ph_3_PAuCl/AgSbF_6_ as catalyst, although the scope was somewhat more limited. The mechanistic pathways of these reactions are under study (Scheme 32) [110].

## Conclusion

In conclusion, in recent years there have been extraordinary advances in the development of gold-catalyzed cycloaddition reactions. The distinctive properties of these metal complexes compared to other, more conventional, transition metal catalysts (e.g., those based on Rh, Ir, Pd, Ru or Ni complexes) and, in particular, their high carbophilicity and ability to stabilize carbocationic intermediates, has allowed researchers to undercover new types of selective and very efficient cycloaddition reactions that would otherwise be unfeasible. Additionally, the scope and versatility of previously reported transition metal-catalyzed cycloaddition reactions (e.g., those based on tungsten or platinum catalysts) could be enhanced by using new gold catalysts. In the future, the current catalogue of cycloaddition reactions catalyzed by gold complexes, and in particular the number of enantioselective versions promoted by chiral gold complexes, is expected to grow.
